# Children’s sedentary behaviour: descriptive epidemiology and associations with objectively-measured sedentary time

**DOI:** 10.1186/1471-2458-13-1092

**Published:** 2013-11-25

**Authors:** Tessa Klitsie, Kirsten Corder, Tommy LS Visscher, Andrew J Atkin, Andrew P Jones, Esther MF van Sluijs

**Affiliations:** 1 Department of Earth and Life Sciences, VU University Amsterdam, Amsterdam, The Netherlands; 2 Medical Research Council, Epidemiology Unit, University of Cambridge, Cambridge, UK; 3 Research Centre for the Prevention of Overweight Zwolle, Windesheim University of Applied Sciences and VU University, Zwolle, The Netherlands; 4 UKCRC Centre for Diet and Activity Research (CEDAR), MRC Epidemiology Unit, University of Cambridge, Cambridge, UK; 5 School of Environmental Sciences, University of East Anglia, Norwich, UK

**Keywords:** Children, Sedentary behaviour, TV viewing, Accelerometer, Socioeconomic status

## Abstract

**Background:**

Little is known regarding the patterning and socio-demographic distribution of multiple sedentary behaviours in children. The aims of this study were to: 1) describe the leisure-time sedentary behaviour of 9–10 year old British children, and 2) establish associations with objectively-measured sedentary time.

**Methods:**

Cross-sectional analysis in the SPEEDY study (Sport, Physical activity and Eating behaviour: Environmental Determinants in Young people) (N=1513, 44.3% boys). Twelve leisure-time sedentary behaviours were assessed by questionnaire. Objectively-measured leisure-time sedentary time (Actigraph GT1M, <100 counts/minute) was assessed over 7 days. Differences by sex and socioeconomic status (SES) in self-reported sedentary behaviours were tested using Kruskal-Wallis tests. The association between objectively-measured sedentary time and the separate sedentary behaviours (continuous (minutes) and categorised into 'none’ 'low’ or 'high’ participation) was assessed using multi-level linear regression.

**Results:**

Sex differences were observed for time spent in most sedentary behaviours (all p ≤ 0.02), except computer use. Girls spent more time in combined non-screen sedentary behaviour (median, interquartile range: girls: 770.0 minutes, 390.0-1230.0; boys: 725.0, 365.0 - 1182.5; p = 0.003), whereas boys spent more time in screen-based behaviours (girls: 540.0, 273.0 - 1050.0; boys: 885.0, 502.5 - 1665.0; p < 0.001). Time spent in five non-screen behaviours differed by SES, with higher values in those of higher SES (all p ≤ 0.001). Regression analyses with continuous exposures indicated that reading (β = 0.1, p < 0.001) and watching television (β = 0.04, p < 0.01) were positively associated with objectively-measured sedentary time, whilst playing board games (β = -0.12, p < 0.05) was negatively associated. Analysed in categorical form, sitting and talking (vs. none: 'low’ β = 26.1,ns; 'high’ 30.9, p < 0.05), playing video games (vs. none: 'low’ β = 49.1, p < 0.01; 'high’ 60.2, p < 0.01) and watching television (vs. lowest tertile: middle β = 22.2,ns; highest β = 31.9, p < 0.05) were positively associated with objectively-measured sedentary time whereas talking on the phone (vs. none: 'low’ β = -38.5, p < 0.01; 'high’ -60.2, p < 0.01) and using a computer/internet (vs. none: 'low’ β = -30.7, p < 0.05; 'high’ -4.2,ns) were negatively associated.

**Conclusions:**

Boys and girls and children of different socioeconomic backgrounds engage in different leisure-time sedentary behaviours. Whilst a number of behaviours may be predictive of total sedentary time, collectively they explain little overall variance. Future studies should consider a wide range of sedentary behaviours and incorporate objective measures to quantify sedentary time where possible.

## Background

Along with physical activity and nutrition, sedentary behaviour is thought to be a risk factor for poor health in both children and adults. A high level of sedentary behaviour has been associated with obesity [1] and is positively associated with insulin resistance and metabolic risk in children [2,3]. Television (TV) viewing, in particular, has been associated with unfavourable body composition, reduced fitness, lower self-esteem and poorer academic performance [1]. Associations between health outcomes and other sedentary behaviours are however less clear [4]. Moreover, to what extent these associations are independent of physical activity remains uncertain, as a negative, albeit weak, association has previously been observed between physical activity and sedentary behaviour in children [5]. Nonetheless, it is recognised that children spend a significant proportion of their time being sedentary [6] and that time spent sedentary should be limited [7].

Sedentary behaviour is difficult to assess accurately due to its complex nature, with a variety of behaviours occurring at different times of day and in multiple locations [8,9]. School-based behaviours are usually pre-determined with little flexibility, probably decreasing the variability in sedentary behaviour. A focus on leisure-time sedentary behaviour therefore appears more useful to inform health promotion. However, most sedentary measures focus on highly visible and prevalent behaviours, such as TV viewing and computer use [8,10]. These screen-based behaviours may fail to fully capture the complexity and diversity of children’s leisure-time behaviour patterns [11,12]. Project STIL (Sedentary Teenagers and Inactive Lifestyles) is one of the few studies that assessed a broad range of leisure-time sedentary behaviours in young people (13–16 years) [13,14]. TV viewing, homework and motorised transport were amongst the five most common sedentary behaviours for both boys and girls. Other common behaviours among girls were sitting and talking, playing musical instruments and looking after pets [14], whereas playing computer/videogames and shopping/hanging out were common amongst boys [13].

Accelerometers are widely used in physical activity research and their applicability to the study of sedentariness is increasingly being explored [8]. Using a 100 counts per minute cut-point, waist worn accelerometry has demonstrated good agreement with activPAL for the assessment of sitting [15] and good to excellent classification accuracy for selected sedentary behaviours in children under controlled conditions [16]. In order to develop effective interventions aimed at reducing overall time children spend in sedentary behaviour, it is important to understand the types of behaviours children engage in and whether any of these are representative of overall time spent sedentary. This is of particular interest to TV viewing, which has been suggested as a key sedentary behaviour [17] and is commonly used as the sole marker of sedentary behaviour. Previous research indicates that TV viewing may be a reasonable marker of overall sedentary time in adult women [18], but not adolescents [11] or children [19].

The aims of this study therefore are 1) to describe the leisure-time sedentary behaviour of 9–10 year old British children, and 2) to identify markers of total sedentary time by studying associations between time spent in 12 sedentary behaviours and objectively-measured leisure-time spent sedentary.

## Methods

### Study and population

The Sport, Physical activity, and Eating behaviour: Environmental Determinants in Young people (SPEEDY) study is a population-based cohort study investigating factors associated with physical activity and dietary behaviour among schoolchildren in the county of Norfolk, United Kingdom. Ethical approval for this study was obtained from the University of East Anglia research ethics committee. The analysis presented here uses baseline data when the children were in school Year 5 (9–10 years old). Full details on participant recruitment, study procedures and sample representativeness for the SPEEDY study have been described elsewhere [20]. Briefly, 92 primary schools were recruited and visited for a measurement session. All attending Year 5 children (N = 3619) were invited to participate, 2064 children provided assent as well as parental consent (57% response rate).

Data were collected between April and July 2007. Trained research assistants visited schools to take physical measurements, administer child questionnaires, fit an accelerometer and distribute a home pack (containing an accelerometer instruction sheet and diary, parent questionnaire and food diary) to each child. Participants were asked to return the home packs and accelerometers to school eight days after the visit.

### Assessments

Sedentary time was assessed over one week with an ActiGraph accelerometer (GT1M, Actigraph LCC, Pensacola, US). Participants wore the monitor on an elastic waistband on the right hip during waking hours, except whilst bathing and during other aquatic activities. The monitors were set to record and were analysed in 5-second epochs. Data were processed using a bespoke programme, MAHUffe (Medical Research Council Epidemiology Unit, Cambridge, UK). Periods of ≥ 10 minutes with continuous zero activity counts and any day with < 500 minutes of valid recording were excluded [21-23]. Participants were included when they had at least 3 valid days of data of which one day was a weekend day (N = 1685). Any data recorded after 11 pm and before 6 am were removed to reduce the potential influence of children deviating from the protocol and wearing the monitor during sleep. To establish the outcome measure of leisure-based minutes spent sedentary, matching the self-reported data, school-time data (9 am-3 pm on weekdays) were excluded. A cut-point of <100 counts per minute was applied to define sedentary activity [15,16].

Leisure-time sedentary behaviours were assessed using a slightly modified version of a child self-report questionnaire, the Youth Physical Activity Questionnaire (YPAQ) [24], which is based on the Children’s Leisure Activities Study Survey (CLASS) [25]. Children reported the frequency and duration of 12 leisure-time sedentary behaviours over the past 7 days (activities included: arts and craft, doing homework, listening to music, playing indoors with toys, playing board games or cards, playing musical instruments, reading, sitting talking, talking on the phone, playing videogames, using the computer or internet, and watching TV or videos). No data on school-based sedentary behaviours was collected. For non-screen behaviours, children reported how many days of the week they performed a sedentary behaviour (never, 1 day, 2–3 days or 4 or more days) and the average duration per day. The same response categories were used for weekday screen-based behaviours, whereas the frequency of weekend screen-based behaviours was reported as 'none’ , '1 day’ , or '2 days’. Weekly duration of screen and non-screen sedentary behaviour, and overall reported sedentary behaviour, is reported. In a reliability study of 41 12–18 year olds [24], the one week test-retest reliability of total sedentary time from this questionnaire was high (α = 0.75), with reliability higher for 12–13 year olds (α = 0.73) compared to 16–18 year-olds (α = 0.61). Participants were excluded if they had missing data for at least one of the sedentary behaviours (N = 144) and if they reported total sedentary time of <60 min/wk or >5340 min/week (N = 59).

As an alternative method of describing the sedentary behaviour data, we categorized all exposure variables. For participants reporting engaging in a particular behaviour, a binary variable denoting low or high levels of participation was derived using a median split (see Additional file 1: Table S2), creating a three-category variable (no, low or high engagement). As the prevalence of no TV or video viewing was low (3%), this variable was split into tertiles.

Age and sex were self-reported. Standardized protocols were used to measure height to the nearest 1 mm (Leicester height measure [Chasmors, Leicester, UK]) and weight in light clothing to the nearest 0.1 kg, using a non-segmental bio-impedance scale (type TBF-300A [Tanita, Tokyo, Japan]). Height and weight were used to calculate body mass index (BMI, kg/m^2^) and standard methods were applied to calculate BMI z-score [26]. Home postcodes were used to determine urban/rural location of participant’s home [27]. Four density profiles were collapsed into a dichotomous variable, with 'city’ and 'town and fringe’ areas classified as urban and 'hamlets and isolated dwellings’ and 'villages’ classified as rural. A composite score was used to represent socioeconomic status, consisting of parent-reported age at leaving full-time education (categorised as ≤16 years or >16 years of age), car ownership (yes or no), and house ownership (rental or own/buying).

### Statistical analysis

Analyses were conducted in Stata SE11 (Stata, College Station, TX). Drop-out analyses and differences in baseline characteristics by sex were investigated using *t* tests or *χ*^2^ tests for continuous and categorical variables. Due to the skewed nature of the self-reported data, Kruskal-Wallis tests were used to determine differences in reported time spent in sedentary behaviours by sex and SES. Multilevel linear regression analysis was used to examine the association between the 12 different sedentary behaviours separately and leisure-based sedentary time derived from the accelerometer, allowing for clustering of children within schools. Sex, SES and valid accelerometer registered time were included as covariates. If multiple behaviours were found to be associated with sedentary time (at p ≤ 0.1), a stepwise manual backward selection procedure was conducted. This p-value was chosen because of the exploratory nature of this study. All relevant variables where entered into a model and removed stepwise starting with the one with the highest p-value, resulting in a final model only including variables associated at p < 0.05. To establish sex differences in associations, sex by behaviour interactions were included in the single models and taken forward to the multiple models if p≤0.1. The analyses were then repeated using the sedentary behaviours as categorical variables.

## Results

A total of 1513 participants (73.3% of the study sample) were included in the analyses after excluding participants with incomplete or invalid accelerometer and YPAQ data. Those excluded from the analyses did not differ from those included on age, sex or BMI z-score, however they were more likely to be of lower SES (p = 0.03).

Table 1 presents the descriptive characteristics of the study sample. No sex differences in anthropometric or demographic variables were observed. Overall, children accumulated an average of 2091.3 (SD: 320.8) minutes of objectively-measured leisure time sedentary time (~35 hrs) per week; girls accumulated more sedentary time than boys.

**Table 1:** Descriptive characteristics of the SPEEDY sample, stratified by sex

	**Overall**	**Boys**	**Girls**	***p-value***
Sex n (%)	1513	678 (44.8)	835 (55.2)	
Age, mean ± SD, y	10.3 ± 0.3	10.2 ± 0.3	10.3 ± 0.3	0.29
BMI z-score, mean ± SD	0.38 ± 1.14	0.42 ± 1.11	0.36 ± 1.16	0.34
*Socioeconomic indicators*				
- Own car, %	95.8	95.2	96.4	0.24
- Own/buying home, %	75.3	75.4	75.3	0.96
- Mother left full-time education at >16 y, %	53.0	53.0	53.0	0.78
- Composite SES, %				0.97
Lowest (score: 0 or 1)	17.4	17.5	17.3	
Middle (score: 2)	38.3	38.0	38.6	
Highest (score: 3)	44.3	44.5	44.1	
Home location, % urban	66.1	63.8	67.8	0.10
*Accelerometer-derived data (all: mean ± SD)*				
- Leisure-time sedentary time (min/wk)	2091.3 ± 320.8	2070.4 ± 335.5	2108.3 ± 307.4	0.02
- Valid leisure-time registered time (min/wk)	3364.1 ± 375.7	3393.5 ± 385.0	3340.2 ± 366.5	0.01
- Proportion of registered time spent sedentary (%)	62.2 ± 6.5	61.0 ± 6.8	63.1 ± 6.0	<0.001

The reported p-value is for sex differences.

*Abbreviations*: *SD* standard deviation, *y* year, *min/wk* minutes per week, *SES* socioeconomic status, *BMI* body mass index.

Table 2 shows the self-reported time spent in the different sedentary behaviours, both overall and stratified by sex. Overall, watching TV/videos was the predominant individual behaviour. Statistically significant sex differences were observed for most sedentary behaviours, with the exception of computer/internet use. In general, girls spent more time in non-screen sedentary behaviour than boys, whereas boys spent more time in screen-based sedentary behaviours. On average, boys spent 57.0% (SD: 22.4) of their reported sedentary time in screen-based behaviours, compared to 44.7% (SD: 21.8) of the time in girls (p < 0.001). Boys were more likely than girls to exceed 2 hours per day of screen-based sedentary behaviour (53.7% versus 33.4%, p = <0.001). Differences in time spent in sedentary behaviours by SES are presented in Additional file 1: Table S1. Differences were only observed for non-screen behaviours, with higher values being reported by those of high SES. However, children from low SES and those from high SES both spent more total minutes sedentary than those of medium SES.

**Table 2:** Median and interquartile range for self-reported leisure-time sedentary behaviours stratified by sex (cells in bold represent the sex with the higher amount of time spent on a particular behaviour, the reported p-value is for sex differences)

	**Boys (N = 678)**	**Girls (N = 835)**	**Overall (N = 1513)**	***p-value***
*Non-screen sedentary behaviour, minutes/week*				
Art & craft	0.0 (0.0-60.0)	**30.0 (0.0-112.5)**	20.0 (0.0-75.0)	<0.001
Doing homework	40.0 (10.0-112.5)	**55.0 (15.0-150.0)**	50.0 (10.0-120.0)	0.02
Listening to music	50.0 (0.0-165.0)	**60.0 (15.0-165.0)**	60.0 (10–187.5)	<0.001
Playing indoors with toys	**53.8 (0.0-175.0)**	30.0 (0.0-125.0)	30.0 (0.0-150.0)	0.002
Playing board games/cards	**0.0 (0.0-60.0)**	0.0 (0.0-30.0)	0.0 (0.0-40.0)	0.009
Playing musical instruments	0.0 (0.0-40.0)	**1.0 (0.0-60.0)**	0.0 (0.0-55.0)	<0.001
Reading	60.0 (10.0-165.0)	**82.5 (25.0-250.0)**	75.0 (20.0-220.0)	<0.001
Sitting talking	30.0 (0.0-110.0)	**55.0 (10.0-150.0)**	45.0 (5.0-150.0)	<0.001
Talking on the phone	10.0 (0.0-37.5)	**15.0 (0.0-62.5)**	10.0 (0.0-55.0)	<0.001
*Screen-based sedentary behaviour, minutes/week*				
Playing videogames	**255.0 (90.0-570.0)**	75.0 (10.0-210.0)	135.0 (30.0-371.0)	<0.001
Using computer/internet	90.0 (15.0-270.0)	70.0 (10.0-210.0)	80.0 (15.0-240.0)	0.07
Watching TV/videos	**360.0 (175.0-755.0)**	270.0 (110.0-600.0)	315.0 (135.0-660.0)	<0.001
*Combined screen and non-screen sedentary behaviour, minutes/week*				
Non-screen	670.0 (333.8-1114.5)	**770.0 (390.0-1230.0)**	725.0 (365.0-1182.5)	0.003
Screen-based	**885.0 (502.5-1665.0)**	540.0 (273.0-1050.0)	700.0 (350.0-1330.0)	<0.001
Total sedentary behaviour	**1657.3 (1070.0-2742.0)**	1422.5 (875.0-2215.0	1547.5 (930.0-2445.0)	<0.001

Weekly objectively-measured leisure-time sedentary time differed by sex (see Table 1) and SES (low (mean minutes ± SD): 2039.2 ± 319.84; middle: 2078.6 ± 302.5; high: 2125.8 ± 328.9; p-value for trend: <0.001).

Table 3 shows the results of the adjusted multilevel linear regression models examining the association between the different sedentary behaviours and objectively-measured sedentary time. Five variables were associated with objectively-measured sedentary time, and taken forward to a multiple regression model. In the fully adjusted model, reported time spent reading and watching TV or videos were positively associated, whereas reported time spent playing board games or cards was negatively associated with objectively-measured sedentary time. Behavioural variables contributed an additional 1% to the explained variance (R^2^) in the outcome over and above that explained in the model comprising confounders only (R^2^ for covariates only model =0.56; final model =0.57). Associations were similar for boys and girls as no statistically significant interactions with sex were observed.

**Table 3:** Association between weekly minutes spent in self-reported sedentary behaviours and accelerometer-derived weekly leisure-based sedentary time (minutes), adjusted for SES, sex and accelerometer wear time (beta coefficient (standard error))

	**Single models**	**Multiple model**
Art & Craft	0.00 (0.04)	-
Doing homework	-0.10 (0.06)^^^	ns
Listening to music	0.03 (0.03)	-
Playing indoors with toys	0.03 (0.02)	-
Playing board games/cards	-0.11 (0.06)^^^	-0.12 (0.06)^*^
Playing musical instruments	0.04 (0.05)	-
Reading	0.11 (0.03)^***^	0.11 (0.03)^***^
Sitting talking	0.05 (0.05)	-
Talking on the phone	-0.05 (0.06)	-
Playing videogames	0.03 (0.02)^^^	ns
Using computer/internet	0.03 (0.02)	-
Watching TV/videos	0.04 (0.01)^***^	0.04 (0.01)^**^

^:p < 0.1; *p < 0.05; **p < 0.01;***p < 0.001; -: not entered in multiple model; ns: not significant in multiple model.

Table 4 shows the results of the analyses with categorized time spent in specific sedentary behaviours. Five variables remained significantly associated with objectively-measured sedentary time in the final adjusted model. Compared with not engaging in the activity, those children reporting high levels of sitting talking and either low or high levels of playing videogames were more sedentary overall. In contrast, those reporting low levels of talking on the phone or using a computer spent less overall time sedentary than those not engaging in these behaviours. Lastly, children in the highest tertile of TV viewing accumulated more objectively-measured sedentary time than those in the lowest tertile. Behavioural variables contributed an additional 1% to the explained variance (R^2^) in the outcome over and above that explained in the model comprising confounders only (R^2^ for final model =0.57). One significant interaction was observed; girls reporting low levels of video game play were more sedentary than those not engaging in the activity (vs. none: 'low’ β = 34.1 (95% CI: 3.5; 64.7), 'high’: 40.0 (-0.4; 79.6)). In boys, the association was much stronger, but only significant for those reporting high levels of playing video games (vs. none: 'low’ 82.2 (-0.7; 163.7), 'high’: 125.9 (50.8; 201.0)).

**Table 4:** Association between categories of weekly minutes spent in self-reported sedentary behaviours and accelerometer-derived weekly leisure-based sedentary time (in minutes) adjusted for SES, sex and accelerometer wear time (beta coefficient (standard error))

	**Categorical exposure (reference: none)**			
	**Single models**		**Multiple model**	
	***Low***	***High***	***Low***	***High***
Art & Craft	22.0 (13.3)	27.5 (17.6)	-	-
Doing homework	9.9 (16.2)	30.8 (16.9)^^^	ns	ns
Listening to music	-0.4 (15.4)	4.8 (15.7)	-	-
Playing indoors with toys	13.5 (14.3)	21.1 (13.9)	-	-
Playing board games/cards	13.0 (12.8)	-9.2 (13.6)	-	-
Playing musical instruments	-4.2 (14.4)	10.4 (13.2)	-	-
Reading	-3.9 (20.9)	28.8 (20.9)	-	-
Sitting talking	51.6 (16.9)	34.2 (15.1)^*^	26.1 (16.3)	30.9 (14.5)^*^
Talking on the phone	-35.1 (16.6)^*^	-17.6 (15.4)	-38.5 (16.2)^*^	-28.7 (16.3)
Playing videogames	42.6 (18.2)^*^	69.0 (19.1)^***^	49.1 (17.4)^**^	60.2 (19.0)^**^
Using computer/internet	-31.1 (14.0)^*^	6.6 (14.7)	-30.7 (14.4)^*^	-4.2 (14.7)
Watching TV/videos^$^	29.6 (15.4)^^^	45.0 (12.6)^**^	22.2 (15.7)	31.9 (13.0)^*^

^$^Due to the low number of children not reporting watching TV/video, tertiles were created with categories 0–180 minutes (reference), 185–505 minutes ('low’) and 510 and higher ('high’).

^^^p < 0.1; ^*^p < 0.05; ^**^p < 0.01;***p < 0.001-: not entered in multiple model; ns: not significant in multiple model.

## Discussion

British children aged 9–10 years engage in a variety of sedentary behaviours, with participation varying by sex and SES. Both sexes reported spending a substantial percentage of their leisure-time engaged in screen-based behaviours, with boys (57.0%) more so than girls (44.7%). Of the non-screen behaviours, most time was spent reading, followed by listening to music and sitting talking. A complex pattern of associations with objectively-measured sedentary time emerged, including both screen and non-screen based behaviours and negative as well as positive associations. However, the magnitude of these associations and the amount of variance in the outcome explained by these variables was too small to conclude that any single behaviour can be considered a meaningful marker of overall sedentary time.

Few studies have been undertaken describing the sedentary behaviours of children that included assessment of multiple screen and non-screen-based behaviours. We found that boys spent more time watching TV/videos, playing indoors with toys, playing board games/cards and playing videogames than girls. In contrast, girls generally spent more time in most non-screen behaviours. Consistent with our findings, other studies found that boys watch significantly more TV and played more videogames [13,14,28,29] than girls and that girls spent more time talking on the phone than boys [13,14]. A study in Chinese children also reported that primary school-aged girls spent more time than boys in activities such as extracurricular reading, writing and drawing [30]. Our observation that boys spent more time in screen-based and total sedentary behaviour than girls, but that girls spent more time in non-screen sedentary behaviours is consistent with findings in 9–16 year old Australians [29]. However, studies exploring sex differences in a range of sedentary behaviours, including non-screen activities, in children of this age are lacking. With specific information on what sedentary behaviours boys and girls do, it may be possible to target interventions more effectively.

We also studied differences by SES, which we defined using a composite score of parental education, car and house ownership. Whereas clear sex differences were observed for most behaviours, only some of the non-screen behaviours differed by SES. Children from a high SES background tended to report more time on non-screen sedentary behaviours although the overall reported time spent sedentary did not differ substantially between children from low and high SES backgrounds. Previous research has also reported higher engagement in non-screen sedentary behaviours among those from high SES backgrounds (as defined by parental education and household income), but also that those from low SES backgrounds spent more time in screen-based sedentary behaviour [29]. Using an area-based measure of deprivation, a recent study showed a similar pattern, but also that those from areas of lower deprivation (e.g. higher SES) tended to accumulate more sedentary time overall [28]. We observed similar patterns with the objectively-measured data. Research into SES differences in physical activity levels have also shown differences according to the type of SES measure used and the type of physical activity assessed [31], which may also partially explain the disparities in findings. Further research into the association between SES and sedentary behaviour is needed to disentangle what components of SES are important for children’s sedentary behaviour.

The common perception of TV viewing as an indicator of overall time spent sedentary was partially supported by this study, adding to a literature with mixed findings on this subject [11,19,32]. A significant association between TV viewing and accelerometer-assessed sedentary time was also observed in a recent European study [19]. However, similar to the current study, the authors concluded that the relationship was weak, as indicated by the magnitude of the association and the limited amount of variance explained. We added to the current evidence by investigating whether sedentary behaviours other than TV viewing might serve as a marker for overall sedentary time. When considering the behaviours as continuous variables, in addition to TV viewing, reporting more reading and less time playing board games or cards were associated with higher objectively-measured sedentary time. Given the skewed nature of the data and the observation that questionnaires are generally more suitable for ranking individuals [24], we also considered the behaviours as categorical variables. Here, a mix of positive and negative associations was again observed, with playing videogames emerging as the behaviour most strongly associated with objectively-measured sedentary time, particularly for boys. In both the continuous and categorical analyses, little additional variance in the outcome was explained over and above that of the confounders, even when multiple behaviours were retained in the final statistical model. Taken together, these results suggest that overall time spent sedentary, as measured by accelerometry, is unlikely to be captured accurately by focusing on a single behaviour.

To our knowledge, this is the first study to provide a descriptive epidemiology of a variety of sedentary behaviours in 9–10 year old children. It utilized both an objective and subjective measure of sedentary behaviour, assessed a wide range of screen and non-screen behaviours and considered differences by sex and SES. The large sample size and heterogeneity in location are key strengths of the SPEEDY study. Several limitations are acknowledged. Accelerometer and YPAQ data did not refer to the same week, with the YPAQ data referring to the week before the accelerometer was worn. Although this might partially explain the lack of associations observed, previous research has shown that children’s overall sedentary time is relatively stable day to day [33]. Although the sedentary items of the YPAQ questionnaire were deemed reliable, no data on the validity is available. However, the aim here was not to obtain a valid estimate of sedentary time per se, but to obtain information on the types of behaviours children engage in and to rank them in terms of participation; in general, questionnaires are an appropriate methodology to achieve this [24]. The SPEEDY data were collected in 2007 and we acknowledge that the media landscape has changed significantly since then, likely affecting the descriptive epidemiology provided. However, it is unlikely to affect the association with objectively-measured sedentary time. Lastly, differential drop-out was observed with children from low socioeconomic backgrounds less likely to provide valid data. The SPEEDY sample has also previously been shown to under represent obese children relative to the broader Norfolk population of this age, limiting the representativeness of the results shown.

## Conclusions

Children engage in a wide variety of sedentary behaviours and participation varies by sex and SES. Interventions that target a single behaviour may be too narrow in focus to impact upon overall sedentary time. Moreover, careful consideration should be given to the selection of intervention targets because the mechanisms linking sedentary behaviour with health outcomes remain unclear [34,35] and not all sedentary behaviours are associated with the same health risks [4]. This study adds to this debate by highlighting that participation in sedentary behaviours that may be considered more or less beneficial, socially or developmentally, varies across the population and individual behaviours may be positively or negatively associated with overall sedentary time. Future studies, both observational and experimental, should consider a wide range of sedentary behaviours and incorporate objective measures to quantify sedentary time where possible.

## Competing interests

The authors declare that they have no competing interests.

## Authors’ contributions

APJ and EMFvS initiated the study and managed data collection. TK, KC and EMFvS formulated the research question and analysis plan, TK analysed the data under supervision of KC and EvS and wrote the initial draft of the manuscript. EMFvS and AJA performed additional analyses and re-drafted the manuscript. All authors contributed to the interpretation of the results and provided input in the revisions of the manuscript. All authors have read and approved the final version of the manuscript.

## Pre-publication history

The pre-publication history for this paper can be accessed here:

http://www.biomedcentral.com/1471-2458/13/1092/prepub

## Supplementary Material

Additional file 1 **Table S1.** Median and interquartile range for self-reported sedentary behaviours described for SES (cells in bold represent the SES category with the highest amount of time spent on that particular behaviour, the reported p-value is for difference by SES). **Table S2.** Descriptive statistics of categories of time spent in sedentary behaviours. Click here for file
