# Supplementary material for: Children’s sedentary behaviour: descriptive epidemiology and associations with objectively-measured sedentary time
Source: BMC Public Health. 2013 Nov 25;13:1092. doi: 10.1186/1471-2458-13-1092 (PMC4222753; doi:10.1186/1471-2458-13-1092)
Supplement: Additional file 1 — Table S1. Median and interquartile range for self-reported sedentary behaviours described for SES (cells in bold represent the SES category with the highest amount of time spent on that particular behaviour, the reported p-value is for difference by SES). Table S2. Descriptive statistics of categories of time spent in sedentary behaviours. [file 1471-2458-13-1092-S1.doc]

**Supplementary table 1.** Median and interquartile range for self-reported sedentary behaviours described for SES (cells in bold represent the SES category with the highest amount of time spent on that particular behaviour, the reported p-value is for difference by SES).

|  | Lowest (N=246) | Middle (N=542) | Highest (N=626) | *p-value* |
| --- | --- | --- | --- | --- |
| *Non-screen sedentary behaviour, minutes/week* | | |  |  |
| Art & craft | 22.5 (0.0-90.0) | 15.0 (0.0-75.0) | 15.0 (0.0-75.0) | 0.67 |
| Doing homework | 35.0 (10.0-110.0) | 45.0 (10.0-112.5) | **60.0 (20.0-150.0)** | 0.001 |
| Listening to music | 75.0 (5.0-300.0) | 60.0 (10.0-165.0) | 55.0 (10.0-165.0) | 0.20 |
| Playing indoors with toys | 15.0 (0.0-137.5) | 30.0 (0.0-150.0) | **52.5 (0.0-150.0)** | <0.001 |
| Playing board games/cards | 0.0 (0.0-40.0) | 0.0 (0.0-40.0) | 0.0 (0.0-40.0) | 0.53 |
| Playing musical instruments | 0.0 (0.0-30.0) | 0.0 (0.0-40.0) | **8.0 (0.0-75.0)** | <0.001 |
| Reading | 55.0 (10.0-275.0) | 60.0 (15.0-165.0) | **90.0 (30.0-261.3)** | <0.001 |
| Sitting talking | 30.0 (0.0-110.0) | 37.5 (2.5-150.0) | **55.0 (10.0-150.0)** | <0.001 |
| Talking on the phone | 12.5 (0.0-60.0) | 10.0 (0.0-55.0) | 10.0 (0.0-50.0) | 0.38 |
| *Screen-based sedentary behaviour, minutes/week* | | | |  |
| Playing videogames | 155.0 (30.0-405.0) | 145.0 (30.0-367.5) | 131.0 (30.0-330.0) | 0.25 |
| Using computer/internet | 60.0 (0.0-255.0) | 90.0 (15.0-252.5) | 90.0 (20.0-210.0) | 0.20 |
| Watching TV/videos | 300.0 (135.0-600.0) | 300.0 (120.0-660.0) | 330.0 (150.0-720.0) | 0.11 |
| *Combined screen and non-screen sedentary behaviour,* *minutes/week* | | | | |
| Non-screen | 755.6 (340.0-1231) | 660.0 (312.5-1095.0) | **770.5 (427.5-1220.0)** | 0.005 |
| Screen-based | 727.5 (380.0-1530.0) | 668.8 (345.0-1312.5) | 705.0 (360.0-1305.0) | 0.62 |
| Total sedentary behaviour | **1586.3 (1005.0-2583.5)** | 1427.5 (837.0-2397.5) | 1572.5 (1025.0-2440.0) | 0.04 |

NOTE: no data on SES was available for 99 participants

**Supplementary table 2:** Descriptive statistics of categories of time spent in sedentary behaviours.

|  | Descriptive statistics | |
| --- | --- | --- |
|  | % none | median minutes |
| Art & Craft | 42.2 | 62.5 |
| Doing homework | 17.1 | 75 |
| Listening to music | 21.7 | 100 |
| Playing indoors with toys | 32.2 | 82.5 |
| Playing board games/cards | 54.0 | 50 |
| Playing musical instruments | 56.0 | 60 |
| Reading | 13.4 | 82.5 |
| Sitting talking | 22.1 | 75 |
| Talking on the phone | 31.5 | 30 |
| Playing videogames | 16.3 | 195 |
| Using computer/internet | 21.5 | 122.5 |
| Watching TV/videos^a^ | 3.4 | 180/505 |

^a^Due to the low number of children not reporting watching TV/video, categorical variable was derived using tertiles (0-180; 185-505; 510+)
